# Circulatory EVs as a predictor of chronic urticarial activity

**DOI:** 10.5339/qmj.2022.fqac.5

**Published:** 2022-03-22

**Authors:** Ramzy Mohammed-Ali, Jayakumar Jerobin, Sally Khalil, Siveen Sivaraman, Manjunath Ramanjaneya, Ilham Betahi, Abdul Badi Abou Samra, Maryam Al-Nesf

**Affiliations:** ^1^Allergy and Immunology Division, Department of Medicine, Hamad Medical Corporation, Doha, Qatar E-mail: JKumar3@hamad.qa; ^2^Qatar Metabolic Institute, Academic Health System, Hamad Medical Corporation, Doha, Qatar; ^3^Translational Research Institute, Academic Health System, Hamad Medical Corporation, Doha, Qatar

**Keywords:** biomarkers, CU pathogenesis, EV

## Abstract

**Background:** Chronic urticaria (CU) is a common and complex disorder that occurs without any identifiable provoking factor. The mechanisms underlying CU pathogenesis are still not fully understood. The autoimmune theory of IgG autoantibodies to IgE/high-affinity receptor of IgE on mast cells and mast cell activation and autoallergy (IgE-mediated disease) might contribute to CU pathogenesis. Extracellular vesicles (EVs) are membranous vesicles released from apoptotic or activated cells of different types. In this study, we aimed to investigate circulating EVs as potential biomarkers in patients with CU compared with that in healthy controls. **Methods:** We studied 15 patients with CU and 16 healthy controls. Circulatory EVs (plasma) were characterized by the presence of externalized phosphatidylserine (annexin V staining). An unpaired t-test was used, and P < 0.05 was considered statistically significant. **Results:** We did not find significant differences in the total number of EVs in patients with CU. A significant decrease in the levels of T-cells (CD3) and endothelial cells (CD146) (P < 0.05) in these patients than in controls was found. No significant differences were observed between patients with CU and healthy controls in terms of platelets, macrophages, PECAM-1, B cells, and tissue factors. **Conclusion:** Endothelial cells have been shown to contribute to the pathogenesis of CU and are also targeted by mediators released by mast cells and other cellular infiltrates. We identified that circulatory endothelial and T-cell EVs might play an important role in CU pathogenesis. In addition, our study highlights the importance of EVs as future therapeutic targets to be investigated.

